# Effectiveness and cost-effectiveness of prognostic markers in prostate cancer

**DOI:** 10.1038/sj.bjc.6600630

**Published:** 2003-01-28

**Authors:** N W Calvert, A B Morgan, J W F Catto, F C Hamdy, R L Akehurst, P Mouncey, S Paisley

**Affiliations:** 1 Fourth Hurdle Consulting Ltd, 2 Fisher Street, London WCIR 4QA; 2 School of Health and Related Research, University of Sheffield, Regent Court, 30 Regent St., Sheffield S1 4DA, UK; 3 Academic Urology Unit, Division of Clinical Sciences, Royal Hallamshire Hospital, Glossop Rd, Sheffield S10, UK

**Keywords:** prostate cancer, prognostic marker, cost-effectiveness, DNA-ploidy

## Abstract

This paper demonstrates how economic modelling can be used to derive estimates of the cost-effectiveness of prognostic markers in the management of clinically localised and moderately graded prostate cancer. The model uses a Markov process and is populated using published evidence and local data. The robustness of the results has been tested using sensitivity analysis. Three treatment policies of ‘monitoring’ (observation), radical prostatectomy, or a selection-based management policy using DNA-ploidy as an experimental marker, have been evaluated. Modelling indicates that a policy of managing these tumours utilising experimental markers has an estimated cost per quality-adjusted life year (QALY) of £12 068. Sensitivity analysis shows the results to be relatively sensitive to quality-of-life variables. If novel and experimental markers can achieve specificity in excess of 80%, then a policy of radical surgery for those identified as being at high risk and conservative treatment for the remainder would be both better for patients and cost-effective. The analysis suggests that a radical prostatectomy treatment policy for the moderately graded tumours (Gleason grades –7) modelled in this paper may be inferior to a conservative approach in the absence of reliable prognostic markers, being both more costly and yielding fewer QALYs.

Prostate cancer is the commonest malignancy in men in the UK. In 1994, there were 20 000 new cases registered in England and Wales, accounting for 15% of all cancers (Office for National Statistics, 2000). The widespread use of prostate-specific antigen (PSA) testing is likely to have significantly increased this figure by 2001. While the clinical course of each tumour is unpredictable, most tumours are slow growing and many men with localised disease live over 10 years after diagnosis (Chodak *et al*, 1994). Accurate staging of prostate cancer is difficult to achieve and up to 45% of tumours are regraded after surgical resection (Steinberg *et al*, 1997; Ross *et al*, 1999). To date, the best prognostic indication is a combination of the clinical stage, Gleason grade, and serum PSA at diagnosis (Partin *et al*, 1997).

PSA testing in previously unscreened healthy younger men (within or out with a screening programme) is increasingly common. The increased incidence of affected younger men and the downstaging effect of PSA testing have coincided with the development of radical local treatments with curative intent on locally confined cancer. More widespread PSA testing therefore is likely to have significant resource implications both in terms of testing and subsequent treatment. The development of one or more prognostic markers that would more accurately predict biological behaviour would enable the rationalisation of radical treatment, and achievement of subsequent health and economic benefits.

As with most human cancers, the era of molecular medicine has brought to the fore potential markers of the biology of each tumour. They have mostly failed to enter the clinical arena. This paper utilises available information on the cell's chromosomal content (ploidy) as a template for modelling the role of prognostic markers in prostate cancer. Ploidy has widely been suggested to predict cancer behaviour (Schroder *et al*, 1994; Ross *et al*, 1999; Bostwick *et al*, 2000; Montironi, 2001). The maintenance of chromosomal balance (diploidy) is vital to a cell's DNA stability and loss is thought to herald a decline in differentiation with imbalance of many oncogenes and tumour suppressor genes. In addition, new automated image-based ploidy analysis machines have been developed to allow rapid, reproducible, and reliable ploidy analysis of tumour samples obtained by needle-core biopsy. We have developed a model to analyse the economic and quality-of-life effects of adding an experimental prognostic marker such as ploidy to the traditional pathway of organ-confined prostate cancer.

## Materials and methods

**Figure 1:**
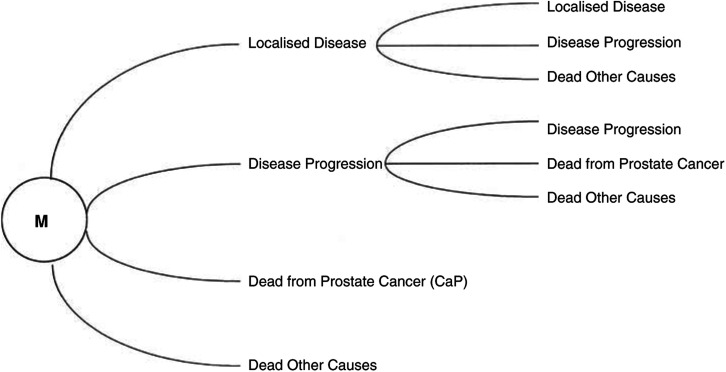
Markov representation of early localised prostate.

A decision analytic Markov model was built using Microsoft Excel™ and populated using evidence from published literature and local data sources. The Markovian structure of the model is represented in Figure 1

. A theoretical cohort of 1000, 60-year-old men diagnosed with moderately differentiated (Gleason sum score 5 – 7) prostate cancer has been modelled, representing the majority of tumours seen in clinical practice (Han *et al*, 2001). The aggressiveness of these moderately graded tumours is particularly difficult to predict in the absence of additional reliable prognostic markers.

The patient cohort is initially assigned to the state of having being diagnosed with, and living with, early localised prostate cancer. Patients either remain in this state (redefined as disease-free for patients undergoing successful surgery), transfer to the aggressive (metastatic) cancer state, or transfer to the end state ‘dead from other causes’. Three transitional states are also defined for those patients who progress to the metastatic disease state. These are: ‘living with metastatic disease’; ‘dead from prostate cancer’; and ‘dead from other causes’. In each model state, patients incur treatment costs and are assigned a quality-of-life utility. Costs and benefits are accrued over the patient's lifetime or until 40 years have elapsed, whichever occurs first. Total and incremental costs and quality-adjusted life years (QALYs) for three treatment policies are calculated. Future costs and benefits are discounted for time preference at a rate of 6% per annum.

Based on the results for a nonscreened Swedish population (Johansson *et al*, 1997), it is assumed that 22% of our modelled cohort has cancers that are ‘true aggressive’ (i.e., they would progress to metastases if untreated). Johansson's data also suggest that for this cohort of the population, the 15-year progression-free survival rate is 6.2% and, that having progressed; the 15-year survival rate is then 5.7%. These rates have been converted to annual event probabilities in the model by assuming exponential survival curves. The remaining 78% of the modelled cohort are deemed to have ‘true localised’ cancers, and are assumed to have a survival function determined by that of 60-year-old UK men adjusted to exclude deaths from prostate cancer (HM Government Actuarial Department, 2001).

Three policy options are modelled:

1. observation (also known as watchful waiting/monitoring) for all;
2. surgical treatment for all (radical prostatectomy);
3. a selection-based policy, using DNA-ploidy as a prognostic marker.

(1) *Monitoring*. Under the observation arm of the model, all patients are assumed to have conservative treatment consisting of PSA tests every 6 months for the first 2 years of treatment, and every 12 months thereafter. These tests continue until the patient dies or progresses to metastatic disease.

(2) *Radical prostatectomy for all*. The ‘radical prostatectomy for all’ policy arm of the model assumes that the entire patient cohort is given a prostatectomy following diagnosis of localised disease. Based on published data (Dillioglugil *et al*, 1997), a 0.5% perioperative death rate is assumed. A total of 25% of ‘true aggressive’ cancers are assumed avoided following prostatectomy (Kattan *et al*, 1997), such cancers then being modelled as if they were ‘true localised’ following successful surgery.

(3) *Prognostic marker-based selective radical intervention policy*. Patients with a diploid test are presumed to have less aggressive disease, and are treated using ‘observation’ in the model. Patients with a nondiploid test are assumed to have more aggressive cancers, and are treated with prostatectomy. Having assumed sensitivity and specificity values for the DNA-ploidy test, and also what proportion of patients are ‘true aggressive’, Bayes theorem was used to determine what proportion of patients would be expected to have a diploid test result. Assuming a test sensitivity of 75% and a specificity of 85%, for example, and a ‘true aggressive’ cancer prevalence of 22%, positive and negative predictive values are calculated to be 0.59 and 0.92, respectively (Table 1

**Table 1:** Relation between sensitivity, specificity, and predictive values*

	**Presumed aggressive**	**Presumed localised**	
True aggressive	0.165(a)	0.055 (b)	0.22 (a+b)
True localised	0.117 (c)	0.663 (d)	0.78 (c+d)
	0.282 (a+c)	0.718 (b+d)	1.0 (a+b+c+d)

* The cell ‘a+b’ indicates prevalence of aggressive cancer (22%). Sensitivity and specificity were set to be 0.75 (a/a+b) and 0.85 (d/c+d), respectively. Positive and negative predictive values were then calculated to be 0.585 (a/a+c) and 0.923 (d/b+d), respectively.

).

### Treatment costs

An NHS costing perspective is taken so that direct patient costs and indirect costs to the economy are ignored. Costs have been inflated in the model to 2000/01 financial year prices using a combination of hospital and community health services (HCSH) and RPI inflators (Netten and Curtis, 2000; The Fairmount Group, 2001). All patients alive and free of metastases are assumed to have two PSA tests for the first 2 years after diagnosis, and annually thereafter. The blood test itself is assumed to cost £6 per test in 1995/96 prices (Chamberlain *et al*, 1997). It is assumed that hospital consultants will want to review patients so that PSA testing takes place during a hospital outpatient visit costed at £58 per outpatient consultation (Netten and Curtis, 2000). The cost of radical prostatectomy is estimated at £4938 using figures provided by a local NHS Trust. This cost is similar to previously published estimates (Chamberlain *et al*, 1997). The costs of DNA-ploidy analysis have been estimated using confidential information provided by an imaging company. The cost estimates that required equipment life and usage estimates with discounting indicate that staff rather than equipment costs dominate the costs of ploidy analysis (£43 compared with £7), resulting in an estimated total cost per test of £50. Patients progressing to metastatic disease are assumed to have hormone treatment administered medically using LH-RH analogues with an annual cost estimate of £1589 per patient, based on BNF prices for Zoladex (Royal Pharmaceutical Society of Great Britain, 2001). This treatment regimen is assumed to continue until death.

### Quality of life

Years spent alive are quality adjusted using utility values where perfect health is represented by a utility score of one, and death is represented by a value of zero. UK data suggest that men over the age of 60 years have utility scores of 0.78 falling to 0.75 by the age of 75 years (Kind *et al*, 1999). These utility scores have been used to represent men assumed to have aggressive cancers removed by prostatectomy with no complications following surgery. Patients surviving radical prostatectomy are assigned an average utility score of 0.70 to reflect the reduced quality-of-life experienced by a proportion of surgery patients through peri- and postoperative complications (notably persistent incontinence and/or impotence). Meta-analyses of complication rates imply that 48% of radical prostatectomy patients become impotent and remain impotent 18 months after surgery, and have an 18% risk of long-term incontinence (Selley *et al*, 1997). Table 2

**Table 2:** Quality-of-life utility assumptions

**Health state**	**Utility**	**95% CI (range)**	**Source**
Living with localised CaP	0.72	0.63–0.80	Cowen *et al* (1996)
UK male aged <75 years cured of aggressive cancer by RP. No complications	0.78		Kind *et al* (1999)
UK male aged 75+ years cured of aggressive cancer by RP. No complications	0.75		Kind *et al* (1999)
Metastatic CaP	0.42	0.33–0.51	Cowen *et al* (1996)
Impotence	0.69	0.61–0.77	Cowen *et al* (1996)
Incontinence	0.57	0.46–0.68	Cowen *et al* (1996)

presents quality-of-life utility values used in the model.

## Results

The central case assumptions described above were modelled. At 15 years, 19% of the untreated cohort is predicted to progress to metastatic disease, and 14% to die of cancer. These results are compatible with Johansson's outcomes given that we have modelled a younger population. Published sensitivity and specificity figures for Gleason grading in predicting aggressive cancers are 73% and 84%, respectively (Veltri *et al*, 1996). The modelled central scenario assumes sensitivity and specificity of 75% and 85%, respectively, for the ploidy marker. The resulting discounted costs and QALYs for the three modelled treatment policies are presented in Table 3

**Table 3:** Modelled cost and QALY outcomes^a^

	**Cost**	**QALYs**	**Inc. cost/QALY**
Observation	£1659051	7095	
Ploidy marker	£2948177	7202	£12068
RP for all	£6389507	7068	Dominated

a Costs and QALYs discounted at 6%.

. The table shows that observation is the least cost policy (£1.6 m), whereas ‘surgery for all’ is considerably more expensive at £6.4 m. The marker-based treatment selection policy yields 107 more QALYs than the observation policy, resulting in an incremental cost per QALY of £12068. ‘Prostatectomy for all’ is dominated by the other two policies; that is, it is both more expensive and yields fewer QALYs.

### Sensitivity analysis

**Figure 2:**
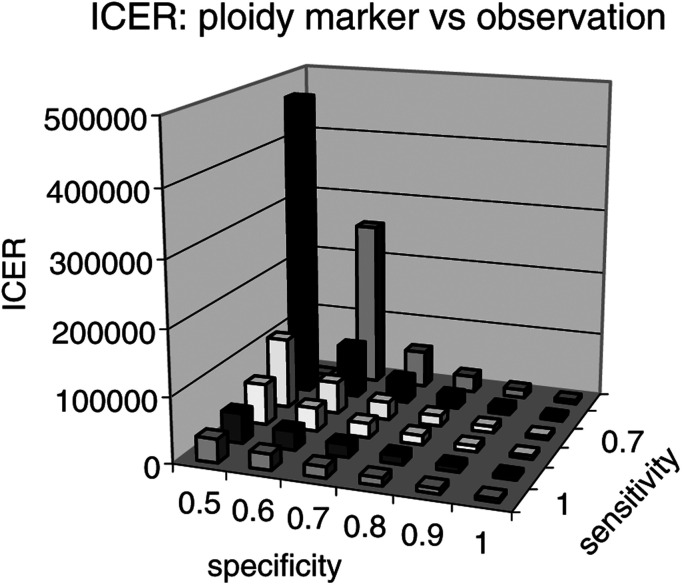
Incremental cost-effectiveness ratio (costs per QALY for marker treatment policy *vs* observation) by test sensitivity and specificity.

Given the lack of published sensitivity and specificity values for ploidy in predicting aggressive prostate cancer, the sensitivity of model outputs was tested by varying sensitivity and specificity between the ranges 50% and 100%. The resulting incremental cost-effectiveness ratios (ICERs) for the marker-based treatment policy compared with observation are depicted graphically in Figure 2

**Table 5:** Table 5

	**Specificity**						
		**0.5**	**0.6**	**0.7**	**0.8**	**0.9**	**1.0**
	0.5	Dominated	255750	56693	25159	12304	5323
	0.6	471520	80259	36513	19673	10755	5232
Sensitivity	0.7	109040	49198	27630	16514	9734	5168
	0.8	63464	36248	22633	14461	9012	5119
	0.9	45613	29145	19429	13019	8473	5081
	1.0	36090	24658	17200	11951	8056	5051

**Figure 3:**
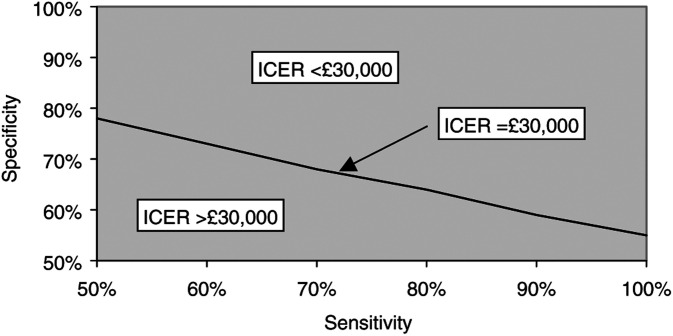
Threshold analysis showing cost per QALY of £30000 for ploidy marker selection policy compared with observation by test sensitivity and specificity.

. Using £30 000/QALY as a threshold range of acceptability for healthcare interventions, modelling indicates that the marker-based selection policy is cost-effective compared with observation for most scenarios. For sensitivity and specificity of 50%, the marker-based policy is dominated by observation (i.e. less effective and more costly). Figure 3

illustrates the threshold combinations of sensitivities and specificities that produce a cost per QALY of £30 000. Combinations above and to the right of the isoquant indicate costs per QALY below £30 000, and *vice versa* below and to the left of the isoquant. The ICER is clearly more responsive to changes in specificity than to sensitivity, and the marker test is cost-effective for all combinations examined when the specificity is 78% or greater. For sensitivity and specificity values tested, the number of QALYs from the marker-based treatment policy never falls below the total achieved in the surgery policy central scenario.

Using a threshold ICER of £30 000/QALY, the sensitivity of the ICERs for the marker-based selection policy with respect to observation, and for the ‘surgery for all’ policy with respect to the marker policy were tested using threshold analysis. The results of the analysis are presented in Table 4

**Table 4:** Threshold sensitivity analyses

**Variable**	**Base case assumption**	**Ploidy marker *vs* observationICER^a^=£30000**	**Surgery *vs* Ploidy marker ICER^a^=£30000^b^**
Sensitivity	75%	34%	NA
Specificity	85%	66%	12%
% True aggressive	22%	11%	67%
% Cured by surgery	25%	15%	NA
15-year progression-free survival	6.2%	30%	NA
15-year disease-specific survival	5.7%	96%	NA
Utility (living with local CaP)	0.72	0.75	0.69
Utility (metastatic)	0.42	0.95	NA
Impotence (Utility)	0.69	0.63	0.76
Impotence (%)	48.4%	82%	10%
Incontinence (Utility)	0.57	0.40	0.76
Incontinence (%)	17.8%	32%	1%
P(peri-op death)	0.5%	43%	NA
Cost of prostatectomy	£4938	£11,730	NA
Cost per ploidy test	£50.26	£1966	£7485
Discount rate	6%	13%	NA

a ICER=incremental cost-effectiveness ratio.

b The shaded boxes highlight those results where a feasible threshold value has been achieved, but where radical prostatectomy is still dominated by the policy of observation.

. The results indicate that the central scenario cost-effective result for the marker policy is pretty robust for all variables except the utility values for quality-of-life when living with the knowledge of having localised prostate cancer, and impotence following prostatectomy. The former needs only to vary from its central value of 0.72 outside the range 0.69–0.75 to render the cost-effectiveness of ploidy doubtful compared to the alternative policies. Likewise, the central value for impotence utility can only vary from 0.69 within the range 0.63–0.76. Table4 indicates that many of the modelled input variables can be changed to their extremes, and still the policy of ‘radical prostatectomy for all’ does not become cost effective compared to the selective marker-based policy. The shaded boxes highlight those results where a feasible threshold value has been achieved, but where radical prostatectomy is still dominated by the observation treatment policy.

## Discussion

This paper has investigated the cost-effectiveness of a selective treatment policy using a prognostic marker (DNA-ploidy) by employing economic modelling techniques. Modelling by definition involves simplification of the real world. The lack of long-term follow-up randomised controlled trial data makes modelling an ideal analytical tool in this context (Fleming *et al*, 1993; Cantor *et al*, 1995; Kattan *et al*, 1997; Yoshimura *et al*, 1998). The model presented is concerned specifically with moderately graded tumours in early stage localised prostate cancers, using DNA-ploidy as a prognostic marker. The results must be viewed in this context; however, the model could be used to model any defined group of tumours and/or prognostic marker. Modelling has indicated that a treatment selection policy based on DNA-ploidy analysis would be cost-effective if the test can achieve specificity levels at or above 80%.

The cost-effective result has been shown to be robust for a range of sensitivity analyses. The ICER is most sensitive to the quality-of-life assumptions for patients living with the knowledge that they have untreated localised prostate cancer. The thresholds required in order to change our cost-effective result lie within reported 95% confidence intervals for this variable (Cowen *et al*, 1996; Kattan *et al*, 1997). Our utility assumption for this variable lies in the range 92%–96% of full health for the age range modelled. Litwin *et al* (2001) have reported that untreated early localised prostate cancer may have lower quality-of-life scores, which would make the marker and ‘prostatectomy for all’ policies more cost-effective using this model. The result is also relatively sensitive both to the frequency and quality-of-life assumptions associated with the modelled post-prostatectomy complications of impotence and incontinence. Our assumptions about the prevalence of impotence and incontinence are consistent with Litwin's data. Reduced levels of complications following surgery, and, or higher utility scores for these complications would again favour the marker-based selection policy and prostatectomy. Further research concerned with guiding treatment policy for early localised prostate cancer should ensure data collection on quality-of-life utility scores for key health states.

Modelling has indicated that for moderately graded early localised tumours, prostatectomy looks relatively expensive with few QALY benefits compared to observation and marker selection-based policies. Some authors have questioned the Johansson data implying that the mortality and progression risks are low (Aus and Hugosson, 1997; Walsh and Brooks, 1997). Higher mortality and progression risks would shift the economic arguments more towards the radical treatment options; however, our sensitivity analysis indicates that these risks would have to increase substantially before our results were invalidated for the central scenario modelled.

Despite the current lack of evidence that screening for prostate cancer prolongs survival and/or improves quality-of-life, PSA screening may well increase in the UK, as men become more aware of prostate cancer and demand PSA testing from their GPs (Donovan *et al*, 2001). Screening implications have not been modelled. Screening a wider population of men than occurs in the UK currently is likely to mean a proportion of men being screened who have no evidence of prostate cancer. This will have cost implications. Also, the introduction of screening in the UK will lead to the identification of earlier stage tumours than are currently being diagnosed. Both the aggressiveness of these tumours and the life expectancies of the screened patients will influence the modelled cost-effectiveness of the available treatment options, and of screening itself. A range of survival and disease progression assumptions could be analysed using this model to help clinicians and patients alike in making difficult treatment decisions in the management of prostate cancer as well as to assess the cost-effectiveness of screening policies.

If novel and experimental markers can achieve specificity in excess of 80% in predicting the aggressiveness of the tumours modelled in this paper, then a policy of radical surgery for those identified as being at high risk, and conservative treatment for the remainder may be both better for patients and cost-effective. The main caveats for the analysis and these conclusions surround quality-of-life issues. There is a clear need to measure reliable utility scores in future research to clarify the QALY benefits of prognostic marker-based treatment policies and prostatectomy in the management of prostate cancer. Models like the one presented in this paper could be used to assess the cost-effectiveness of other prostate cancer patient populations and alternative prognostic markers.
